# Reproducibility of GMP-compliant production of therapeutic stressed peripheral blood mononuclear cell-derived secretomes, a novel class of biological medicinal products

**DOI:** 10.1186/s13287-019-1524-2

**Published:** 2020-01-03

**Authors:** Maria Laggner, Alfred Gugerell, Christiane Bachmann, Helmut Hofbauer, Vera Vorstandlechner, Marcus Seibold, Ghazaleh Gouya Lechner, Anja Peterbauer, Sibylle Madlener, Svitlana Demyanets, Dirk Sorgenfrey, Tobias Ostler, Michael Erb, Michael Mildner, Hendrik Jan Ankersmit

**Affiliations:** 1 Division of Thoracic Surgery, Medical University of Vienna, Vienna, Austria; 2 Aposcience AG, Vienna, Austria; 3 Independent Consultant, Mainaschaff, Germany; 4 Austrian Red Cross Blood Transfusion Service of Upper Austria, Linz, Austria; 5 Molecular Neuro-Oncology, Department of Pediatrics and Adolescent Medicine and Institute of Neurology, Medical University of Vienna, Vienna, Austria; 6 Comprehensive Cancer Center of the Medical University of Vienna, Vienna, Austria; 7 Department for Laboratory Medicine at the Medical University of Vienna, Vienna, Austria; 8 Dr. Regenold GmbH, Badenweiler, Germany; 9 SYNLAB Analytics and Services Switzerland AG, Birsfelden, Switzerland; 10 Research Division of Biology and Pathobiology of the Skin, Department of Dermatology, Medical University of Vienna, Vienna, Austria

**Keywords:** Regenerative medicine, Cell-free secretomes, Paracrine factors, Secretome, Good manufacturing practice, Reproducibility, ICH criteria, Biological medicinal products, Specifications

## Abstract

**Background:**

The recent concept of secretome-based tissue regeneration has profoundly altered the field of regenerative medicine and offers promising novel therapeutic options. In contrast to medicinal products with a single active substance, cell-derived secretomes comprise pleiotropic bioactive ingredients, representing a major obstacle for reproducible drug product efficacy and warranting patient safety. Good manufacturing practice (GMP)-compliant production guarantees high batch-to-batch consistency and reproducible efficacy of biological medicinal products, but different batches of cellular secretomes produced under GMP have not been compared yet, and suitable quality control parameters have not been established. To this end, we analyzed diverse biological and functional parameters of different batches produced under GMP of the secretome obtained from γ-irradiated peripheral blood mononuclear cells with proven tissue regenerative properties in infarcted myocardium, stroke, spinal cord injury, and skin wounds.

**Methods:**

We quantified key secretome ingredients, including cytokines, lipids, and extracellular vesicles, and functionally assessed potency in tube formation assay, ex vivo aortic ring sprouting assay, and cell-based protein and reporter gene assays. Furthermore, we determined secretome stability in different batches after 6 months of storage at various ambient temperatures.

**Results:**

We observed that inter-batch differences in the bioactive components and secretome properties were small despite considerable differences in protein concentrations and potencies between individual donor secretomes. Stability tests showed that the analytical and functional properties of the secretomes remained stable when lyophilisates were stored at temperatures up to + 5 °C for 6 months.

**Conclusions:**

We are the first to demonstrate the consistent production of cell-derived, yet cell-free secretome as a biological medicinal product. The results from this study provide the basis for selecting appropriate quality control parameters for GMP-compliant production of therapeutic cell secretomes and pave the way for future clinical trials employing secretomes in tissue regenerative medicine.

## Background

Stem cells (SCs) are characterized by the ability to differentiate into various cell types and are capable of homing to injured sites [1–5]. Thus, the use of SCs seems to be an attractive and promising approach for tissue regeneration. However, the initial idea of transdifferentiating SCs for tissue repair [6] was refuted by more recent studies, which reported that SC integration into damaged tissues is poor [7] and that SC-derived paracrine factors rather than transplanted SCs themselves are responsible for functional restoration of injured tissues and organs [8, 9]. The efficacy of SC-derived secreted factors was later demonstrated in various pre-clinical studies [10–13], and the use of cell-free therapeutic agents is encouraged in numerous indications (reviewed in [14–19]). Using cell-free paracrine factors exhibit several advantages compared to cell-based therapeutics and resolve major safety considerations of cell transplantation. The application of secretomes is considered to be safer than administering viable, proliferating cells [20], as secretomes lack self-replicating entities and, therefore, harbor no tumorigenic potential. While allogeneic cell transplantation often elicits immune reactions [21], cell-free secretomes are immune-compatible and largely deprived of immunogenic cell surface protein expressions and, therefore, do not require administration of immunosuppressive agents. Furthermore, sterile filtration of cellular secretomes reduces the risk of biological contamination and can be performed without substantial loss of efficacy [22]. Nonetheless, SC plasticity, loss of stemness during in vitro manipulation [23], and the generally limited accessibility of SCs complicate large-scale and affordable production for the therapeutic application of SC secretomes.

We previously showed that infusion of stressed peripheral blood mononuclear cells (PBMCs) suspensions inhibits tissue damage in a rodent model of acute myocardial infarction (AMI) [24]. In 2011, we reported that the ability to secrete regenerative factors is not unique to SCs and that secretomes from SCs and stressed PBMCs exhibit comparable regenerative effects [25]. We have also demonstrated that injecting secretome alone is sufficient to improve cardiac outcome and reduce infarct area in a rodent and a porcine AMI model [26]. These findings were corroborated in latent porcine model of chronic post-myocardial infarction [27], and comparable tissue regenerative effects were observed in rodent models of cerebral ischemia, acute spinal cord injury, and skin wounds treated with the secretome of stressed PBMCs (PBMC^sec^) [28–30], further highlighting the versatile therapeutic potential of cell-free secretomes obtained from irradiated PBMCs in various indications.

In the past few years, numerous modes of action have been attributed to PBMC^sec^, including immunomodulatory, cytoprotective, vasodilatory, pro-angiogenic, and anti-microbial effects [31]. Stressed PBMCs secrete a variety of bioactive substances, including proteins, peptides, different lipid species, and extracellular vesicles (EVs) [26, 30, 32], which account for the observed effects. We previously showed that the concerted action of all ingredients, including EVs, proteins, and lipids, is required to exert the pro-angiogenic effects ascribed to PBMC^sec^, whereas no single fraction accounts for the full potency of the secretome [26, 30, 32]. These findings justify the use of secretome as a whole rather than purified biomolecules to exploit the full therapeutic effect of PBMC^sec^. When comparing PBMCs and PBMC subsets, we found that the compositions of PBMC subpopulation secretomes are distinct from PBMC^sec^, and pro-angiogenic potency is more pronounced in PBMC^sec^ than subset secretomes [33]. We also observed that γ-irradiation induces necroptosis in the majority of PBMCs rather than apoptosis. Indeed, chemical inhibition of necroptosis, but not apoptosis, results in diminished pro-angiogenic potential of PBMC^sec^, suggesting that cellular necroptosis is an indispensable prerequisite for conferring regenerative, pro-angiogenic properties to PBMC^sec^ [33]. Taken together, our previous data demonstrated the necessity of utilizing the entirety of secreted factors instead of purifying certain classes of biomolecules, using PBMCs rather than specific cellular subpopulations, and stressing cells with γ-irradiation to induce necroptosis in order to exploit the full regenerative capacity of PBMC^sec^.

By definition, biological medicinal products contain one active biological substance obtained from a biological source, such as cells or tissues, which has been biotechnologically manipulated. In contrast to biological medicinal products with one active ingredient like hormones or antibodies, cellular secretomes comprise a pleiotropic mix of diverse components that, in most cases concertedly, mediate pharmacological effects. Advanced therapy medicinal products (ATMPs) represent another medicinal substance class and cover three types of medicinal products: gene therapy medicinal products, somatic cell therapy medicinal products, and tissue-engineered products (Regulation EC No 1394/2007). This definition clearly defines recombinant nucleic acids, manipulated cells or tissues, and engineered cells or tissues for regeneration as ATMPs. However, cell-free secretomes are unambiguously not categorized as ATMPs. From a regulatory point of view, cell-derived secretomes therefore fall within a normative gap and, strictly speaking, cannot be appropriately assigned to the standard classification for biologicals currently employed by regulatory authorities. Despite this discrepancy in definitions, cell-free secretomes are to date categorized as biological products due to the lack of a more suitable classification system.

Defining and controlling the complex composition of all secretome ingredients in full detail is often not feasible, if not impossible. To compensate for the “partial” product characterization, a validated production process and tight control thereof are necessary to guarantee product efficacy, safety, reproducibility, and quality. Good manufacturing practice (GMP) ensures the consistent and quality-controlled production of pharmaceuticals. Despite adhering to strict manufacturing guidelines, inter-donor differences potentially cause large batch-to-batch variability of cellular secretomes. Therefore, assuring reproducibility is crucial for drug product quality and, eventually, marketing authorization for secretomes. In addition to a controlled manufacturing process, a pre-defined set of physico-chemical-biological tests, collectively referred to as product specification criteria, represents a crucial part of the control strategy to ensure the efficacy and consistency of secretomes as biological products. The selection of appropriate tests occurs in a product-specific manner, and specifications are linked to the hypothesized mode of action related to a certain indication. The documented requirements that products have to fulfill for release and the rationale for the range of acceptance criteria are established by the manufacturer based on data obtained during product development, the characterization, manufacturing consistency, manufacturing process development, pre-clinical studies, and stability tests. Specifications may cover several aspects, including physicochemical properties, the quantities of selected constituents, and potency. Specifically, pharmacopeias provide guidance for product evaluation, and analytical procedures for biological products are advised, such as tests for sterility, uniformity of dosage units, and moisture content for lyophilized drug products. Furthermore, qualitative descriptions of the physical state, color of the lyophilisate, and color and clarity of the reconstituted lyophilisate should be given. General tests often entail physical assessments of other quality attributes, such as pH and osmolality. In addition, specifications include functional tests that determine the biological properties of a product to mediate a desired pharmacological action. The ability to achieve a defined biological effect is determined by valid and validated potency assays, often by employing cell culture-based methods, which detect specific cellular responses functionally related to relevant pharmaceutical effects of the biological product. Suitable control parameters need to be sufficiently sensitive to detect potential differences due to the variability of donor material and due to changes in product quality. Results are calibrated against (inter-) national reference standards, when available, or, in lack thereof, against an in-house reference standard appropriately characterized by the manufacturer.

The ability of a biological medicinal product to exert certain biological effects declines over time due to physical or chemical degradation mechanisms. Therefore, the International Council for Harmonization (ICH) provides guidelines for testing the stability of biological products, which are intended to demonstrate that the drug product meets its specification throughout its intended shelf life (ICH Q1A R2). For biological products, a single stability-indicating test is usually not sufficient to assess the stability profiles of all components, and the set of stability tests is product-specific by testing factors relevant for the proposed mode of action and should be sensitive to changes in product quality.

Reproducible and GMP-compliant manufacturing of cell-free secretomes as biologicals has not been reported thus far. Therefore, we sought to compare different PBMC secretome batches produced under GMP to assess the consistency, potency, and stability of a pleiotropic biological product. As PBMC^sec^ contains more than a single active ingredient, a number of physico-chemical and biological parameters and functional assays have been selected to reflect key regenerative components and functions that mediate several of the observed biological activities.

## Materials and methods

### Quality statement

Aposcience AG (Vienna, Austria), Austrian Red Cross Blood Transfusion Service of Upper Austria (Linz, Austria), SYNLAB Analytics and Services Switzerland AG (Birsfelden, Switzerland), and Symbiosis GmbH (Eppelheim, Germany) have validated quality management systems. All tests were carried out and documented according to validated standard operating procedures (SOPs).

### PBMC^sec^ manufacturing

**Fig. 1:**
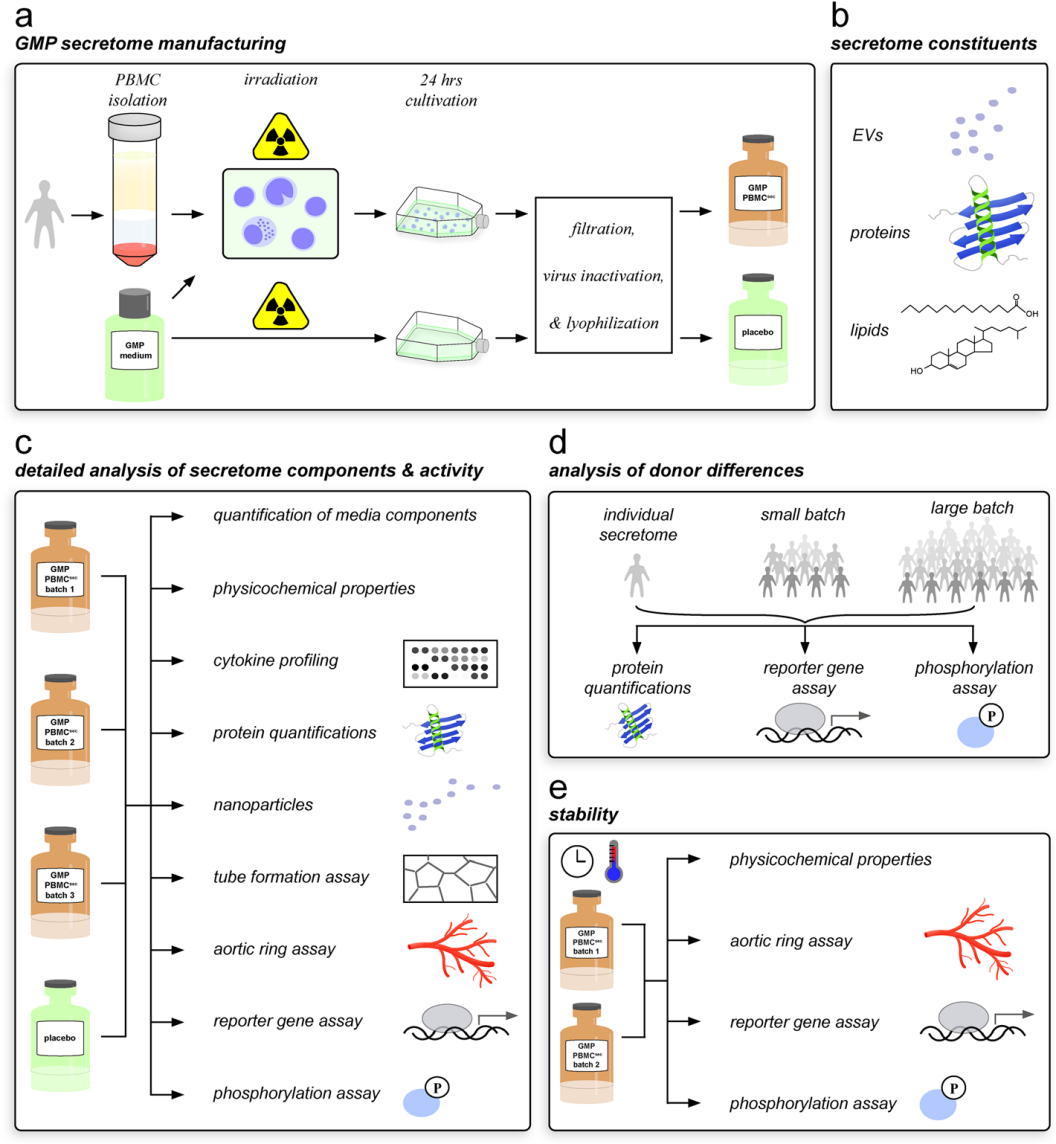
Experimental setup. **a** PBMCs were enriched by Ficoll-assisted centrifugation, exposed to 60 Gy γ-irradiation, and cultured in GMP medium for 24 h. Secretome was filtered, virus-inactivated, and lyophilized (GMP PBMC^sec^). To produce placebo control, GMP medium alone without cells was treated equally. **b** Major biomolecular components present in PBMC^sec^ (EVs, proteins, and lipids) were analyzed. **c** A set of physico-chemical-biological tests reflecting key effects mediated by PBMC^sec^ was selected to determine the reproducibility of 3 PBMC secretome batches produced under GMP. In parallel, tests were performed with one placebo batch. **d** Selected proteins were quantified and functional assays were performed to compare variability of individual donor secretomes, small, and large secretome batches. **e** Stability of 2 PBMC^sec^ batches was assessed after storage at various ambient temperatures for up to 6 months. EVs, extracellular vesicles; GMP, good manufacturing practice; P, phosphorylation; PBMC^sec^, secretome of γ-irradiated, peripheral blood mononuclear cells

PBMC^sec^ were produced in compliance with GMP by the Austrian Red Cross Blood Transfusion Service for Upper Austria (Linz, Austria) as described previously [29]. Briefly, PBMCs were enriched by Ficoll-assisted centrifugation, exposed to 60 Gy Cesium 137 γ-irradiation (IBL 437C, Isotopen Diagnostik CIS GmbH, Dreieich, Germany), and adjusted to a concentration of 2.5 × 10^7^ cells/mL in CellGenix GMP DC medium (CellGenix, Freiburg, Germany) (Fig. 1a). After 24 ± 2 h of culture, cells and cellular debris were removed by centrifugation and conditioned medium containing the secretome was passed through a 0.22-μm filter and virus-inactivated by methylene blue-assisted blue light treatment. To obtain small batches, the secretomes of 12 donors were pooled. For final production scales of large batches (approximately 2.5 L), the secretomes of 96–120 donors from small batches were combined, lyophilized, and terminally sterilized by high-dose γ-irradiation. In the current study, the following batches were analyzed: A000918399131 (designated PBMC^sec^ 1), A000918399150 (PBMC^sec^ 2), and A000919399165 (PBMC^sec^ 3). CellGenix medium processed like PBMC^sec^ without cells (A000918399114) served as placebo control. Lyophilisates were routinely stored at − 80 °C.

### Analysis of medium components

Quantification of selected components present in culture medium was performed by the Department for Laboratory Medicine at the Medical University of Vienna (Vienna, Austria; ISO 15189-accredited) using assays validated for whole blood (Fig. 1c). Albumin, cholesterol, and triglycerides were quantified by ALB2 assay, CHOL2 assay, and TRIGL assay, respectively, using a Cobas 8000 modular analyzer (all Roche Diagnostics, Basel, Switzerland) according to the manufacturer’s instructions.

### Physicochemical parameters

The appearance of the lyophilisate, dissolution time, color, clarity and degree of opalescence, water content, pH, osmolality, uniformity of mass, and presence of visible particles served as physicochemical parameters of the PBMC secretome and were determined by Symbiosis GmbH (Eppelheim, Germany) (Fig. 1c). Tests were performed as recommended by European Pharmacopoeia (Ph. Eur.) analytical standards (9th edition). The pH was determined according to Ph. Eur. 2.2.3 potentiometric determination of pH. For color determination, Certipur reference color solutions green yellow (GY) 1–7 (Merck, Burlington, MA, USA) were used (Ph. Eur. 2.2.2 degree of coloration of liquids). Formazin turbidity standard [4000 nephelometric turbidity units (NTU)] reference solutions (Hach Company, Loveland, CO, USA) were used to determine the degree of opalescence (Ph. Eur. 2.2.1 clarity and degree of opalescence of liquids). The water content of lyophilized secretome was assessed using sodium tartrate dihydrate hydranal standard (Honeywell Fluka, Honeywell International Inc., Morris Plains, NJ, USA) according to the Karl Fischer titration (Ph. Eur. 2.5.12 semi-micro determination of water). A calibration standard of 2000 mosmol/kg (Gonotec GmbH, Berlin, Germany) was used to determine osmolality (Ph. Eur. 2.2.35 osmolality).

### Cytokine profiling

Cytokines present in the secretome were assessed using the Proteome Profiler Assay Human XL Cytokine Array Kit (R&D Systems, Minneapolis, MN, USA) as suggested by the manufacturer (Fig. 1b, c). The intensities of chemiluminescent signals were determined for cytokines with signals above background noise. The mean gray values of duplicate cytokine dots of gray-scale images were determined using the ImageJ measure tool (v1.50i, National Institutes of Health, Bethesda, MD, USA) [34].

### Protein quantification

Proteins crucial for immunomodulation and remodeling of extracellular matrix components and peptides with antimicrobial activity were selected for detailed quantification [35, 36]. Concentrations of interleukin-8 (IL-8), transforming growth factor beta 1 (TGFβ1), and epidermal growth factor (EGF) were measured by the Blood Transfusion Service for Upper Austria, and the amounts of matrix metallopeptidase 9 (MMP9) and plasminogen activator inhibitor 1 (PAI-1) were determined by SYNLAB Analytics and Services Switzerland AG using enzyme-linked immunosorbent assays (ELISAs) (Human EGF Coated ELISA Kit, Human IL-8 Coated ELISA kit, Human MMP-9 Coated ELISA Kit, and Human PAI-1 Coated ELISA Kit, all Invitrogen, Carlsbad, CA, USA; Human TGF-β1 Quantikine ELISA, R&D Systems). S100A8/S100A9 heterodimer (calprotectin) was quantified by ELISA (R&D Systems) according to the manufacturer’s protocols (Fig. 1b, c). All tests were performed according to standard procedures following good laboratory practice (GLP).

### Nanoparticle measurements

Effects and molecular constituents of EVs present in PBMC^sec^ were reported previously [30, 32]. Nanoparticles were isolated and reconstituted in phosphate-buffered saline (PBS) as described previously [30] (Fig. 1b, c). Qualitative and quantitative assessments of nanoparticles were performed by the Molecular Neuro-Oncology, Department of Pediatrics and Adolescent Medicine and Institute of Neurology, Medical University of Vienna (Vienna, Austria), using the NanoSight NS500 instrument (Malvern Instruments, Malvern, UK). For each measurement, 500 μL of the diluted sample was loaded by the automatic pump control into the NanoSight system. The repetition and duration of captures were set manually (5 captures with 30 s each) and used for all measurements. The instrument was calibrated using 100-nm-particle reference controls provided by the instrument manufacturer.

### Tube formation assay

To compare pro-angiogenic properties of PBMC^sec^, a tube formation assay was performed with human umbilical vein endothelial cells (HUVECs, passage 6) (Fig. 1c). Cells were cultured in endothelial cell growth basal medium-2 (EBM-2; Lonza Group AG, Basel, Switzerland) supplemented with endothelial cell growth medium-2 (EGM-2; BulletKit, Lonza). Prior to the tube formation assay, cells were maintained in EBM-2 containing 2% (vol/vol) heat-inactivated fetal bovine serum (Lonza) overnight and starved in basal EBM-2 for 4 h. Cells were seeded on growth factor-reduced Matrigel Matrix (Corning Inc. Life Sciences, Tewksbury, MA, USA) in μ-slides Angiogenesis (ibidi GmbH, Graefelfing, Germany) at a density of 10^4^ cells/cm^2^ and stimulated with the supernatant obtained from 4 × 10^6^ PBMCs for 3 h. Micrographs were acquired by an inverted phase-contrast microscope (CKX41 Olympus Corporation; Tokyo, Japan) equipped with a × 10 objective (CAch N, 10x/0.25 PhP; Olympus) using a SC30 camera (Olympus) and cellSens Entry software (version 1.8; Olympus). Tubule formation was quantified by the Angiogenesis Analyzer plugin of ImageJ using default settings [37].

### Aortic ring sprouting assay

In addition to tube formation, the pro-angiogenic potential of PBMC^sec^ was functionally tested by a sprouting assay using murine thoracic aortas [29, 30] with minor modifications (Fig. 1c). Briefly, aortic rings were sandwiched in fibrin matrices and cultured with PBMC^sec^-conditioned medium equivalent to the supernatant of 4 × 10^6^ PBMCs. Brightfield micrographs were acquired using an inverted Olympus IX83 scanning microscope (Tokyo, Japan) with cellSens Imaging Software (Olympus, Tokyo, Japan) after culturing explants for 6 days. Outgrowth areas were determined by the ImageJ measure tool [34].

### Potency protein assays

The potencies of different PBMC^sec^ batches were compared by validated, GLP-compliant potency assays performed by Synlab [30, 33] (Fig. 1c). Activator protein 1 (AP-1) promoter activity and heat shock protein 27 (HSP27) phosphorylation were determined by reporter gene assay and ELISA, respectively, as described elsewhere [30, 33]. Final data were normalized to internally established PBMC^sec^ in-house reference batches and are given as relative potency. For PBMC^sec^ 1 and 2, A000918399093 was used as a reference solution, and PBMC^sec^ 3 potency was normalized to A000918399150. The comparability of A000918399093 (small batch) and A000918399150 (large batch) was confirmed.

### Stability measurements

Stability was determined using physicochemical parameters, potency, and aortic ring assays (Fig. 1e). Secretomes were produced by the Blood Transfusion Service for Upper Austria and shipped to Symbiosis GmbH on dry ice for storage under stable conditions. Stability tests were conducted by Symbiosis GmbH and Synlab after 1, 3, and 6 months of storage at − 20 °C (− 30 to − 15 °C), + 5 ± 3 °C, + 25 ± 2 °C with 60 ± 5% relative humidity, and + 40 ± 2 °C with 70 ± 5% relative humidity. For aortic ring stability tests, lyophilized secretomes were reconstituted and stored at the indicated temperatures for 5 days.

### Statistical analyses

Data were analyzed using one-way analysis of variance (ANOVA) with Dunnett’s multiple comparisons post hoc tests (Graphpad Prism version 6.05, GraphPad Software, Inc., La Jolla, CA, USA) and given as arithmetic means ± standard deviations (SDs) with *p* < 0.05 considered statistically significant. Percent deviations were determined by SD/mean × 100.

## Results

### Quantification of media components

First, we investigated the ingredients present in the medium used for culturing PBMCs and compared their quantities between secretome batches (Fig. 1c). For our analyses, we focused on albumin and included the most prominent lipid species, cholesterol and triglycerides. Results revealed little variability between PBMC^sec^ batches (0.6 to 5.2% deviation) and were not remarkably different from placebo (Table 1). These data show that cholesterol, triglycerides, and albumin found in PBMC^sec^ were derived from CellGenix GMP DC medium and were not sequestered by PBMCs. Furthermore, we were able to show that the concentrations of key media components remain unaltered during secretome production.

**Table 1:** Quantification of albumin and selected lipid species in PBMC secretome and placebo

Sample	Albumin (g/L)	Cholesterol (mM)	Triglycerides (mM)
PBMC^sec^ 1	6.8	8	3.0
PBMC^sec^ 2	6.8	8	2.9
PBMC^sec^ 3	6.7	8	3.1
Placebo	6.8	9	2.9
Mean PBMC^sec^ batches	6.8	8.25	2.9
SD PBMC^sec^ batches	0.04	0.4	0.08
% deviation PBMC^sec^ batches	0.6	5.2	2.8

### Physicochemical parameters

To obtain general drug substance information, physicochemical properties were assessed in three different PBMC^sec^ batches and one placebo (Fig. 1c) (Table 2). No discoloration of the PBMC^sec^ lyophilisates was observed, and the water content after lyophilization ranged between 2.6 and 3%. No visible particles or color deviations were observed in any of the reconstituted lyophilisates tested. The color appearance of all solutions resembled green yellow standard 5, and opalescence of all PBMC^sec^ was consistently below reference standard 1. Osmolality was highly comparable between all samples, and dissolution time ranged from 38 to 65 s. All solutions were slightly alkaline with pH values between 8.4 and 8.6, presumably due to the vacuum conditions during lyophilization. Similar results were obtained when analyzing the placebo control (Table 2). These data show that physicochemical properties of lyophilisates and reconstituted lyophilisates are comparable between PBMC secretome batches produced under GMP.

**Table 2:** Physicochemical parameters of lyophilized and reconstituted drug substance and placebo

Test	PBMC^sec^ 1	PBMC^sec^ 2	PBMC^sec^ 3	placebo
Appearance of lyophilisate	White, no color deviations or discolorations, amorphous, homogenous	White, no color deviations or discolorations, amorphous, homogenous	White, no color deviations or discolorations, amorphous, homogenous	White, no color deviations or discolorations, amorphous, homogenous
Dissolution time	65 s	45 s	38 s	34 s
Color of reconstituted solution	Between reference solution GY5 and GY6	Between reference solution GY5 and GY6	Equivalent to reference solution GY5	Between reference solution GY5 and GY6
Clarity and degree of opalescence	Clear, less than reference suspension 1 (3 NTU)	Clear, less than reference suspension 1 (3 NTU)	Clear, less than reference suspension 1 (3 NTU)	Clear, less than reference suspension 2 (6 NTU)
Water content	2.6%	2.6%	3.0%	2.2%
pH	8.4	8.6	8.6	8.8
Osmolality	1.4 osmol/kg	1.4 osmol/kg	1.4 osmol/kg	1.4 osmol/kg
Visible particles	0 against white background, 0 against black background	0 against white background, 0 against black background	0 against white background, 0 against black background	0 against white background, 0 against black background

### Evaluation of protein secretion

Next, we compared cytokine levels between different batches of PBMC^sec^ by membrane immune-detection array (Fig. 1c) (Additional file 1: Figure S1a,b). A comparable cytokine signature was observed throughout batches, which was markedly distinct from the placebo control (Additional file 1: Figure S1c). Detailed results of positive chemiluminescent signals are provided in Table 3.

**Table 3:** Average chemiluminescent signal intensities

Analyte	PBMC^sec^ (mean gray values)	Placebo (mean gray values)
Apolipoprotein A-I	163 ± 24	11 ± 0
BAFF	161 ± 22	12 ± 0
CD14	134 ± 36	19 ± 2
Chitinase 3-like 1	220 ± 20	9 ± 0
EGF	*95 ± 20*	*12 ± 1*
emmprin	124 ± 37	28 ± 1
ENA-78	112 ± 60	9 ± 1
Endoglin	166 ± 27	51 ± 0
IFN-γ	84 ± 9	66 ± 3
IL-8	*192 ± 28*	*8 ± 0*
Kallikrein 3	95 ± 40	79 ± 4
Lipocalin-2	162 ± 11	4 ± 0
MIF	149 ± 11	7 ± 0
MMP-9	*150 ± 22*	*3 ± 0*
Myeloperoxidase	153 ± 10	91 ± 0
Osteopontin	105 ± 22	69 ± 5
PDGF-AA	174 ± 29	13 ± 2
pentraxin 3	174 ± 9	165 ± 2
PF4	133 ± 29	4 ± 0
RANTES	149 ± 27	16 ± 0
RBP4	132 ± 55	115 ± 2
Resistin	113 ± 60	27 ± 0
PAI-1	*255 ± 0*	*12 ± 0*
Thrombospondin-1	119 ± 14	3 ± 0
uPAR	147 ± 60	9 ± 1
Vitamin D BP	138 ± 25	68 ± 5
CD31	130 ± 5	5 ± 0

Cytokines selected for subsequent ELISA-assisted quantifications are highlighted in italics

*BAFF* B cell activating factor, *CD14* cluster of differentiation 14, *EGF* epidermal growth factor, *ENA-78* epithelial-derived neutrophil-activating protein 78, *IFN-γ* interferon gamma, *IL-8* interleukin-8, *MIF* macrophage migration inhibitory factor, *MMP9* matrix metallopeptidase 9, *PDGF-AA* platelet-derived growth factor subunit A, *PF4* platelet factor 4, *RANTES* regulated and normal T cell expressed and secreted, *RBP* retinol binding protein, *PAI-1* plasminogen activator inhibitor-1, *uPAR* urokinase-type plasminogen activator receptor, *vitamin D-BP* vitamin D-binding protein, *CD31* cluster of differentiation 31

Based on data obtained from cytokine profiling, we proceeded to select several bioactive proteins (IL-8, MMP9, EGF, PAI-1, TGFβ1, and, in addition, calprotectin) for protein quantification, as these factors were considered to be at least partly responsible for immunomodulation and extracellular matrix remodeling during wound healing, and exert antimicrobial activity (Fig. 1c). While low variation (~ 10% deviation) was observed when assessing EGF, PAI-1, MMP9, and calprotectin concentrations, these proteins were not detectable in placebo (Table 4). Because sample pooling, lyophilization, and terminal sterilization of PBMC^sec^ may adversely affect protein concentrations and stability, we also determined the concentrations of PAI-1 and MMP9 in the secretomes of individual PBMC donors prior to these manufacturing steps (Fig. 1d). Surprisingly, PAI-1 was highly variable between donors, and concentrations were remarkably decreased by drug substance processing (around 10-fold concentration decrease of individual secretomes compared to pooled secretomes) (Additional file 1: Figure S2a). MMP9 concentrations exhibited equally large variation between individual donors (Additional file 1: Figure S2b), but remained predominantly stable and comparable to amounts detected in final PBMC^sec^ large batches (Table 4). As final production scales were obtained after pooling small batches consisting of 12 donor secretomes each, we furthermore compared PAI-1 and MMP9 concentrations between small and large PBMC^sec^ batches. Though small batches still had variable protein concentrations, large batch concentrations were equivalent to the median of small batches (Additional file 1: Figure S3), indicating that pooling 100 donor secretomes diminishes the high inter-donor differences in PAI-1 and MMP9. Batch-to-batch variability of TGFβ1 and IL-8 was relatively high between large batches (~ 30% deviation; Table 4), yet, variability between small batches was even higher. These results show that pooling secretomes of ~ 100 donors for one large batch compensates certain inter-donor differences, yet particular proteins can still exhibit considerable variability between batches.

**Table 4:** Protein quantification in large PBMC secretome batches. *< LOD* below limit of detection

Sample	PAI-1 (ng/mL)	MMP9 (ng/mL)	EGF (pg/mL)	TGFβ1 (ng/mL)	IL-8 (ng/mL)	Calprotectin (ng/mL)
PBMC^sec^ 1	1.98	438	113	4.75	3.41	31
PBMC^sec^ 2	1.64	639	89	2.25	3.87	27
PBMC^sec^ 3	1.44	600	100	2.79	1.68	29
Placebo	< LOD	< LOD	< LOD	< LOD	< LOD	< LOD
Mean PBMC^sec^ batches	1.69	559	100.7	3.26	2.98	29
SD PBMC^sec^ batches	0.23	87	9.8	1.08	0.94	1.4
% deviation PBMC^sec^ batches	13.3	15.6	9.7	33.0	31.6	4.9

### Evaluation of secreted vesicles

**Fig. 2:**
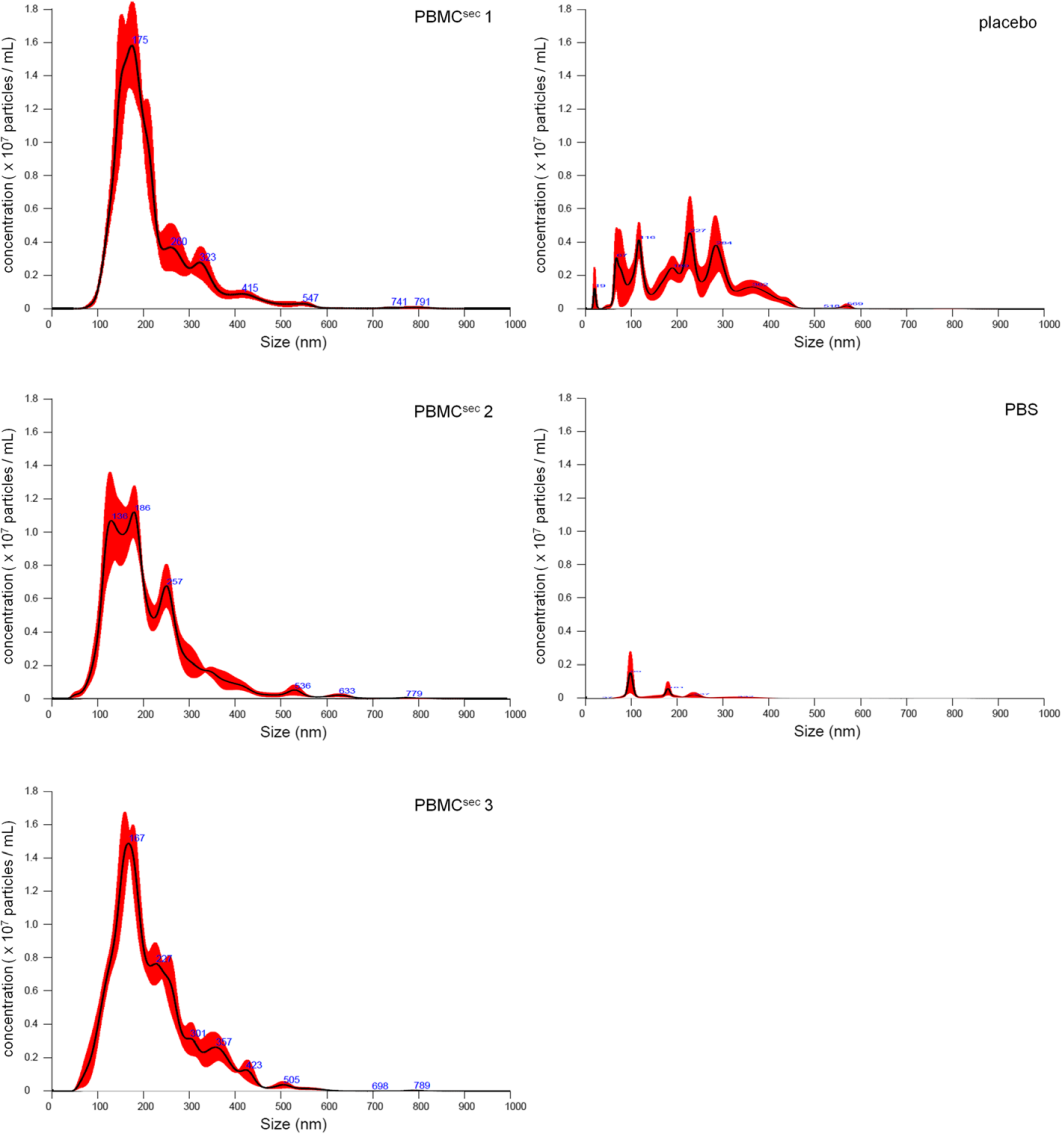
Nanoparticles detected in different batches of PBMC^sec^, placebo, and PBS. The sizes and amounts of nanoparticles detected in PBMC secretomes were compared to particles present in placebo

EVs present in secretomes have gained increasing attention in regenerative medicine, and the molecular composition and effects of PBMC secretome-derived EVs have been described extensively. As a crucial, biologically active component of cellular secretomes, we compared the number and size of EVs present in PBMC secretomes (Fig. 1c). NanoSight measurements revealed high similarity in terms of quantity and quality (Fig. 2). Deviations in particle concentrations and sizes were less than 5% and 0.3%, respectively (Table 5). In placebo, the number of vesicles was less than half the number detected in secretomes. Low levels of particles were detected in PBS used to reconstitute isolated vesicles from large batches. Taken together, these data indicate that GMP-compliant production of PBMC^sec^ allows the reproducibility of vesicle secretion by PBMCs.

**Table 5:** Quantification of extracellular vesicles in PBMC^sec^

Sample	Concentration (particles/mL)	Mean particle size (nm)
PBMC^sec^ 1	1.8 × 10^9^ ± 1.58 × 10^8^	215.6 ± 3.4
PBMC^sec^ 2	1.72 × 10^9^ ± 6.75 × 10^7^	214.2 ± 5
PBMC^sec^ 3	1.93 × 10^9^ ± 8.93 × 10^7^	215.3 ± 3.7
Placebo	7.27 × 10^8^ ± 7.78 × 10^7^	225.9 ± 17.9
PBS	5.34 × 10^7^ ± 1.45 × 10^7^	171.2 ± 20
Mean PBMC^sec^ batches	1.82 × 10^9^	215.03
SD PBMC^sec^ batches	8.65 × 10^7^	0.6
% deviation PBMC^sec^ batches	4.76	0.28

### Functional comparisons

**Fig. 3:**
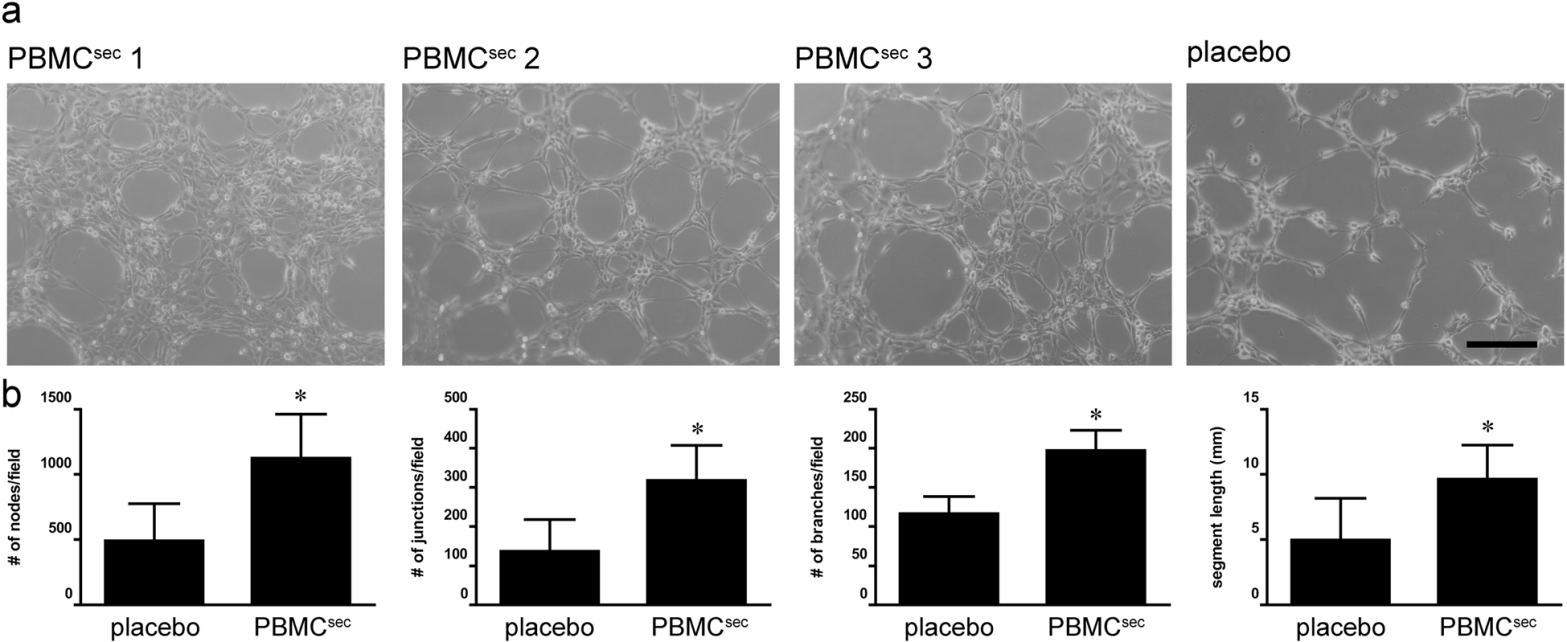
Tube formation assay. **a** Representative images of HUVECs incubated with different secretome batches or placebo. Scale bar, 200 μm. **b** Numbers of nodes, junctions, and branches and segment lengths determined in HUVECs incubated with secretomes or placebo. Bars indicate arithmetic means and error bars indicate SD. *p < 0.05 versus placebo

**Fig. 4:**
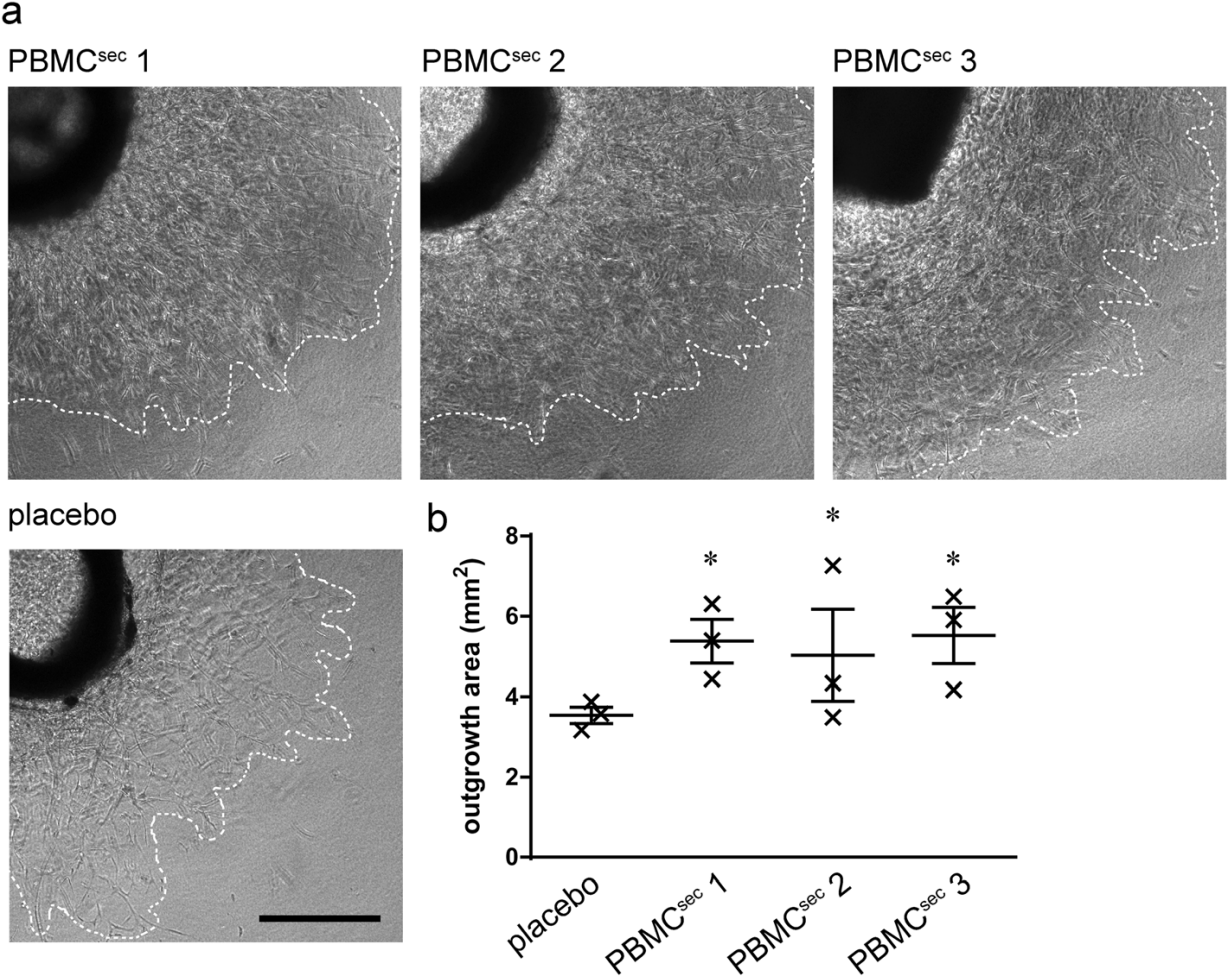
Endothelial cell sprouting assay using murine aortic rings. **a** Representative micrographs of aortas incubated with different secretome batches or placebo. Scale bar, 500 μm. **b** Statistical analysis of sprouting areas. Large horizontal lines indicate medians, and whiskers denote SD. *p < 0.05 versus placebo

In addition to physicochemical parameters and biological ingredients of secretomes, we functionally evaluated drug substance potency (Fig. 1c). As induction of blood vessel formation represents a key event during secretome-promoted wound healing and tissue regeneration, the pro-angiogenic potential of different PBMC^sec^ batches was compared by tube formation and aortic ring sprouting assays, and induction of wound healing-related actions was assessed by AP-1 reporter gene assay and phosphorylation of HSP27. All secretome batches strongly induced tube formation in HUVECs, whereas placebo exhibited negligibly small tube induction potential (Fig. 3). These results were corroborated by findings obtained from murine aortic ring assays, in which blood vessel sprouting consistently improved with PBMC^sec^ compared to placebo (Fig. 4). When assessing AP-1 promoter activity and HSP27 phosphorylation, the potency of PBMC^sec^ was as high as that detected in established reference batches (Table 6). Secretome potency was comparable between all batches tested, with little deviation (0.5–6%), whereas placebo exhibited no detectable potential in potency assays. To further determine the donor variance of secretome potencies, AP-1 promoter activity and HSP27 phosphorylation assays were performed with individual secretomes (Fig. 1d). Between individual donors, we observed up to 2-fold and 4-fold differences in the ability to induce AP-1 and to phosphorylate HSP27, respectively (Additional file 1: Figure S4). When comparing small pools of 12 donors to large batches with 100 donors, pooling compensated for large inter-donor differences (Additional file 1: Figure S5). The data presented here indicate that the pro-angiogenic potency, though considerably different from donor-to-donor, was highly consistent between batches with GMP-compliant production.

**Table 6:** Relative potency detected in different PBMC^sec^ batches. *< LOD* below limit of detection

Sample	AP-1 (%)	p-HSP27 (%)
PBMC^sec^ 1	101	101
PBMC^sec^ 2	100	100
PBMC^sec^ 3	101	87
Placebo	< LOD	< LOD
Mean PBMC^sec^ batches	100.7	96
SD PBMC^sec^ batches	0.5	6.4
% deviation	0.5	6.6

### Assessment of secretome stability

Lastly, we determined drug substance stability by assessing physicochemical parameters and drug substance potency after 3 and 6 months of storage at different ambient temperatures and humidity (Fig. 1e). Physicochemical properties remained consistently stable for up to 6 months when storing lyophilisates between − 20 and + 25 °C (Tables 7 and 8). After storage at + 40 °C for 3 months, the solubility of secretomes was reduced, thereby affecting the assessment of several parameters.

**Table 7:** Physicochemical properties of PBMC^sec^ 1 and 2 after 3 months of storage at various temperatures

Test	− 20 °C	+ 5 °C	+ 25 °C	+ 40 °C
Appearance of lyophilisate	White, no color deviations or discolorations, amorphous, homogenous	White, no color deviations or discolorations, amorphous, homogenous	White, no color deviations or discolorations, amorphous, homogenous	Orange, stained in different shades of orange, several small red spots, amorphous, not homogenous
Dissolution time	53.5 ± 1.5 s	50 ± 5 s	51 ± 8 s	Could not be completely dissolved within 15 min
Color of reconstituted solution	Equivalent to reference solution GY5	Equivalent to reference solution GY5	Equivalent to reference solution GY5	Not equivalent to any of the reference solutions GY1–GY7, color was estimated to be in the range of dark orange
Clarity and degree of opalescence	Clear, equivalent to reference suspension 1 (3 NTU)	Clear, equivalent to reference suspension 1 (3 NTU)	Clear, less than reference suspension 1 (3 NTU)	Between reference suspension 2 (6 NTU) and reference suspension 4 (30 NTU)
Water content	2.65 ± 0.15%	3.1 ± 0.1%	3.7 ± 0.2%	6.15 ± 0.25%^(1)^
pH	8.01 ± 0.51	8.7 ± 0	8.85 ± 0.05	7.95 ± 0.55^(1)^
Osmolality	1.4 ± 0 osmol/kg	1.4 ± 0 osmol/kg	1.4 ± 0 osmol/kg	1.3 ± 0 osmol/kg^(1)^
Visible particles	0 against white background, 0 against black background	0 against white background, 0 against black background	0 against white background, 0 against black background	Visible particles could not be evaluated due to large residues of undissolved sample material

^(1)^As the samples could not be dissolved completely, the validity of these results is compromised

**Table 8:** Physicochemical properties of PBMC^sec^ 1 and 2 after 6 months of storage at different temperatures

Test	− 20 °C	+ 5 °C	+ 25 °C	+ 40 °C
Appearance of lyophilisate	White, no color deviations or discolorations, amorphous, homogenous	White, no color deviations or discolorations, amorphous, homogenous	White, no color deviations or discolorations, amorphous, homogenous	Reddish brown, uniformly stained, amorphous, not homogenous at the edge of the lyophilisate
Dissolution time	40 ± 5 s	36.5 ± 0.5 s	52.5 ± 10.5 s	Could not be completely dissolved within 15 min
Color of reconstituted solution	Equivalent to reference solution GY5	Equivalent to reference solution GY5	Equivalent to reference solution GY5	Not equivalent to any of the reference solutions GY1–GY7, color of solution and undissolved material was estimated to be in the range of yellow to reddish brown^(1)^
Clarity and degree of opalescence	Clear, equivalent to reference suspension 1 (3 NTU)	Clear, equivalent to reference suspension 1 (3 NTU)	Between reference suspension 1 (3 NTU) and reference suspension 2 (6 NTU)	Parameters could not be evaluated due to large residues of undissolved sample material
Water content	4.39 ± 0.12%	4.59 ± 0.10%	5.46 ± 0.09%	7.33 ± 0.43%^(1)^
pH	n.d.	n.d.	8.7 ± 0	7.09 ± 0.04 ^(1)^
Osmolality	n.d.	n.d.	1.4 ± 0 osmol/kg	Parameter could not be evaluated due to large residues of undissolved sample material
Visible particles	n.d.	n.d.	0 against white background, 0 against black background	Visible particles could not be evaluated due to large residues of undissolved sample material

*n.d.* not determined

^(1)^As samples could not be dissolved completely, the validity of these results is compromised

**Fig. 5:**
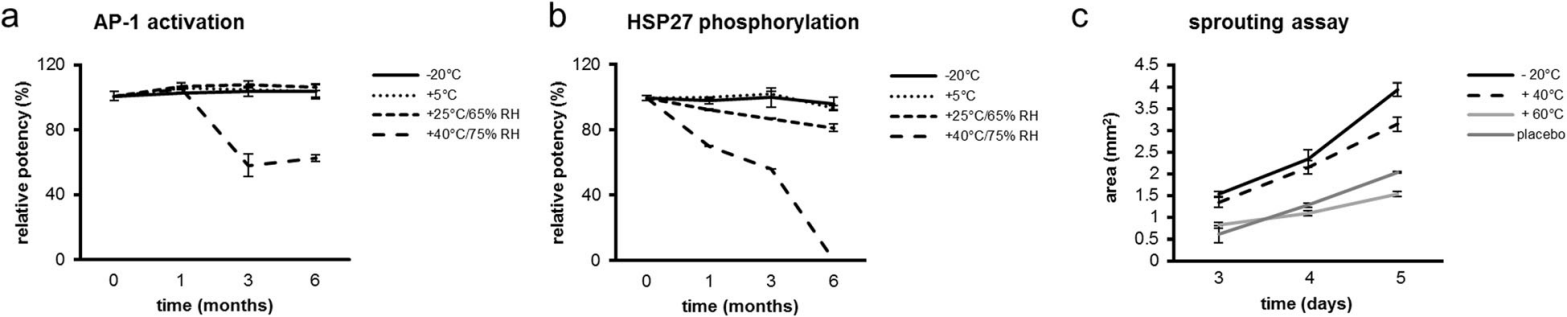
Secretome potency after long-term storage at different temperatures. **a** Ability of PBMC^sec^ to induce AP-1 promoter and **b** phosphorylate HSP27 after storage of lyophilisate at − 20 °C, + 5 °C, + 25 °C, and + 40 °C. **c** Potency of reconstituted lyophilisates stored at different temperatures to induce endothelial sprouting. Lines connect arithmetic means, and error bars indicate SD

The ability of the secretome to induce the AP-1 promoter remained unaltered when lyophilized drug substance was stored at − 20 °C, + 5 °C, and + 25 °C for up to 6 months, whereas storing lyophilisates at + 40 °C caused a decline in AP-1 potency to 58% and 62% after 3 and 6 months, respectively (Fig. 5a). Comparably, the potency of HSP27 phosphorylation remained unaltered when storing lyophilisates at − 20 °C and + 5 °C (Fig. 5b). After 3 months of storage at + 25 °C and + 40 °C, HSP27 potency declined to 87% and 56%, respectively. The HSP27 phosphorylating capacity declined to 81.3% and was undetectable after 6 months’ storage temperatures of + 25 °C and + 40 °C, respectively (Fig. 5b), indicating limits of secretome stability.

We also tested secretome stability by aortic ring assay. To this end, lyophilized secretomes were reconstituted and stored in a liquid state at temperatures ranging from − 20 to + 60 °C for 5 days. Areas of endothelial cell outgrowth were determined on days 3, 4, and 5 of explant culture. Maintaining reconstituted PBMC^sec^ at + 40 °C only marginally decreased the pro-angiogenic potential compared to storage at − 20 °C (Fig. 5c). In contrast, the sprout-inducing ability was remarkably compromised when storing reconstituted secretome at + 60 °C for 5 days, similar to that observed in placebo-treated aortas. Taken together, our data show that storing PBMC^sec^ lyophilisates at temperatures up to + 5 °C for 6 months and reconstituted secretomes below + 40 °C for 5 days does not significantly compromise secretome properties and potency.

## Discussion

The application of cellular secretomes in clinical settings represents an attractive therapeutic approach for treating a large number of diseases and injuries. Though the safety of cell suspensions has been reported previously [38], use of conditioned media may be superior compared to the application of cells. In contrast to cells, cell-free secretomes harbor no tumorigenic potential and terminal sterilization (e.g., by high-dose γ-irradiation) can be performed [39]. Thus, secretomes exhibit advantages that contribute to increased patient safety. Moreover, secretomes may be provided as off-the-shelf products. Toxicity studies for subcutaneous administration of PBMC^sec^ have already been conducted in which subcutaneous injection of PBMC^sec^ was well tolerated [40]. With this in mind, use of cell-free secretomes may be preferable to using cells for therapeutic approaches whenever feasible.

Diverse biological substances present in secretomes mediate pleiotropic effects and account for multiple modes of action [31]. However, drug product specifications and regulatory requirements are a major obstacle for clinical application of cell secretomes, as secretomes are classified as biological medicinal products. Therefore, extensive product characterization and generation of a multifaceted panel covering analytical and functional parameters are a prerequisite for drug product specification and, eventually, allowing the release of secretomes for clinical use. Previously, the reproducibility of different GMP-compliant, human cell-derived secretomes has not been investigated. In the current study, we have quantified several key components and evaluated the biological activity of cellular secretomes of different batches produced in compliance with GMP. We were able to show that batches produced under GMP consistently met their specifications as determined by physicochemical and functional tests. Protein measurements of cytokines and antimicrobial peptides were in line with previously described protein quantifications in PBMC^sec^ [26, 35]. Moreover, our nanoparticle measurements and potency assay results were in accordance with data reported by Wagner et al. [30]. Lastly, we provide evidence of secretome stability when storing lyophilisates below + 5 °C for 6 months. Therefore, we were able to show comparability within secretomes analyzed in this study and between batches produced under GMP investigated here and previous secretomes. The results reported in our study may help establish and select appropriate specification parameters for therapeutic secretomes.

Thus far, no consensus culture protocol is available for promoting the most effective release of regenerative factors, and no agreement yet exists on the most potent source for secretory cells. In contrast to our irradiation, the vast majority of studies employ secretomes from cultured cells without additional stimulation or challenge [10, 12, 13]. However, a limited number of reports have already demonstrated superior effects when manipulating SCs in vitro to promote increased release of regenerative factors. For example, adherent cells can be expanded in spheroid cultures instead of two-dimensional growth patterns, affecting the anti-inflammatory properties of cells [41]. Furthermore, hypoxic culture conditions have been shown to exert beneficial effects on SCs, as indicated by preserved stemness and increased proliferation [42], and administering culture media from hypoxia-preconditioned SCs improved the outcome in animal models of traumatic brain injury and hepatectomized mice compared to secretomes obtained under normoxic conditions [43, 44]. Another approach for in vitro manipulation is stimulation with pro-inflammatory agents [45]. Though promoting regenerative actions of secretomes, these approaches have certain downsides compared to our γ-irradiated PBMCs. In their studies, both Chang et al. [43] and Lee et al. [44] employed 25-fold concentrated secretome from hypoxia-challenged cells, whereas our secretome concentration remained unaltered after reconstitution of the lyophilisate. As induction of necroptosis was reported as an indispensable prerequisite for the pro-angiogenic properties of PBMC^sec^ [33], it remains to be determined whether hypoxic pre-conditioning is equally effective as γ-irradiation in inducing necroptosis in PBMCs. Stimulating cells with pro-inflammatory mediators is disadvantageous, as these agents are then part of the secretome and may cause unwanted reactions when applied in humans. In comparison, γ-irradiation does not require externally added stimulatory agents or drug vehicles and is superior to the addition of any immunomodulators. Therefore, our validated manufacturing process represents a major step towards the application of PBMC^sec^ in clinical studies.

For three different batches of PBMC^sec^ produced under GMP, ELISA-assisted quantification of IL-8 and MMP9 was consistent with results obtained by cytokine profiling. In ELISA, EGF concentrations were comparably high in PBMC^sec^ 1 and 3, and lower levels of EGF were detected in PBMC^sec^ 2. In comparison, the proteome profiler assay revealed the highest amount of EGF in PBMC^sec^ 1, and lower levels in 2 and 3. These minor quantitative discrepancies can be explained by the different methodological approaches. Though ELISA is considered a reliable, well-recognized quantitative assay in which sample signals are quantified using external standard curves, profiler arrays allow for qualitative screening rather than quantitative statements. Thus, for secretome-based drug product specifications, ELISA-assisted quantifications are preferable.

Individual donor secretomes displayed considerable variability of MMP9, PAI-1, and potency. The reduction in variability is reciprocal to the square root of pooled samples and reduced variability after pooling was confirmed. Though variations in PAI-1 and MMP9 concentrations, AP-1-inducting ability, and HSP27-phosphorylating capacity were relatively high in individual donors while being low in large batches, IL-8 and TGFβ1 exhibited larger variability, even after combining up to100 individual secretomes. These data show that pooling several donor secretomes is a crucial step for producing cellular secretomes with comparable composition. Nonetheless, combining secretomes of several donors is an essential safety aspect, which is taken into consideration in the strict donor selection criteria and several in-process controls during the manufacturing process. Though single proteins exhibit a certain degree of variability between large batches, the (more relevant) biological (pro-angiogenic) activity was comparable. To reliably determine drug substance consistency, functional assays are more meaningful than individual secretome constituents, but presumably not sufficient. Moreover, drug substance variability may be decreased by implementing in-process controls, by which unsuitable donors and intermediates may be identified and excluded whenever appropriate, contributing to further optimization of the manufacturing process.

Our functional assays (AP-1 promoter activity, HSP27 phosphorylation, tube formation, and aortic ring sprouting assay) showed equal potential for different PBMC^sec^ batches, and PBMC^sec^ consistently exhibited improved potency compared to placebo. These findings are in accordance with data reported previously [27, 30]. While AP-1 promoter activation and HSP27 phosphorylation represent validated assays involving cell lines with standardized experimental conditions and settings, sprouting assays are based on murine aortas and are subject to more biological variability. Several factors, especially the age of the mice, adversely affect the proliferation of endothelial cells [46] and tissue isolation and processing unfavorably impact cellular viability due to lack of oxygen supply following euthanization [47]. As a result of the methodical complexity and biological variability, aortic ring-based sprouting assays do not represent a feasible read-out for large-scale product specification of cellular secretomes. Using simpler approaches with fewer influential factors and confounders, such as in vitro reporter gene and phosphorylation assays, is therefore preferable for determining the consistency of cellular secretome potency.

Cell culture media are usually limited for research use only and are rarely intended for clinical application. During preparation of therapeutically used cells, culture media required for production are commonly removed by several washing steps prior to application in humans. Nonetheless, certain medium constituents can be adsorbed or internalized and metabolized and may be present in the drug product. In addition, the composition of the medium is altered as ingredients are incubated at 37 °C during the culture period, whereby temperature-sensitive ingredients are potentially degraded. Since the secretome cannot be separated from the culture medium, the medium used to culture PBMCs is part of the final product. With or without media-removal production steps, media and media remnants as part of secretomes thus become a safety issue entailing new quality-related and regulatory issues. Suitability of culture media for clinical use requires thorough risk management, and the quality of the media therefore needs to be carefully assessed in the course of incoming goods inspections. With regard to PBMC^sec^, we performed toxicology studies with repeated intravenous and subcutaneous application of the secretome containing medium in two animal models, which was well tolerated [40]. Moreover, a phase I safety clinical trial with topical application of PBMC^sec^ was successfully performed (ClinicalTrials.gov Identifier: NCT02284360), where safety and tolerability were demonstrated and no therapy-related, serious adverse events were observed [48]. We therefore consider our medium-containing secretome toxicologically safe and with acceptable risk.

Specification criteria for release are highly product-specific, and depending on the cellular source, each secretome requires its own specifications. Nonetheless, parameters reflecting general information, i.e., related to the dosage form, are the same for many drug products, such as physicochemical characteristics and microbiological safety testing. In our study, we determined several identity and potency parameters, namely PAI-1, MMP9, EGF, TGFβ1, IL-8, AP-1 activity, and HSP27 phosphorylation, adding valuable parameters to the list of potential specifications. The suitability of each parameter to adequately reflect drug product efficacy and quality, however, remains to be determined. Further aspects relevant for product release are several safety issues which come along with using human cell-derived secretomes. For instance, the presence of endotoxins and potential allergic reactions to serum-derived proteins have to be taken into consideration. Endotoxin tests are part of the release tests, while indicating the presence of allergens is part of the investigator’s brochure and mentioned in the patient information. Patients with suspected allergies can thus be excluded in advance to prevent occurrence of adverse reactions. Another crucial parameter to be considered for product characterization and specifications are impurities as they represent an inevitable part of medicinal products. In general, impurities should be controlled at the incoming goods control level with a risk evaluation for each component. Impurities may not be determined if they pose negligible risks, e.g., due to low concentrations or because they pose no health risk. In our case, processes like PBMC isolation and virus inactivation potentially contribute to impurities present in the final product, such as anticoagulants, lymphocyte separation medium, or methylene blue. While most reagents are removed by rinsing or centrifugation, trace amounts of methylene blue remain detectable in spite of a depletion step. However, these concentrations are low compared to the amounts patients receive, e.g., during plasma transfusions. Since allergic reactions to methylene blue treatments have been reported, too high concentrations of methylene blue in the final product represent an exclusion criterion. As safety and tolerability of PBMC secretome have already been demonstrated in our toxicology study [40] and our phase I clinical trial, we consider the presence of methylene blue and other impurities of our secretome as low risk. Taken together, the most appropriate and most meaningful parameters reflecting identity, potency, purity, and safety of each secretome may be determined by extensive drug product characterization and, optionally, after consultation with responsible authorities.

As the composition of a cellular secretome is very complex, the entirety of all biomolecules can never be assessed. Therefore, certain classes of biologically active substances are missing in our study, such as miRNAs. The exact molecular composition and related biological processes of miRNAs secreted in EVs by stressed PBMCs have been analyzed previously [30]. Since the regenerative capacity was most pronounced in the entire secretome compared to EVs containing miRNAs [30], we believe that miRNAs contribute little to the pro-angiogenic ability that we assessed. We cannot rule out, however, that miRNAs present in PBMC^sec^ (encapsulated in vesicles or free) mediate other pharmacologically relevant events. Elucidating potential effects caused by miRNAs and further secretome ingredients not determined within this study remains subject of future investigations.

The number of clinical trials employing conditioned media is currently on the rise, highlighting the great regenerative potential of secretomes. These future studies will eventually reveal the true applications for secretomes in regenerative medicine.

## Conclusions

Taken together, we demonstrated the reproducible identity, potency, and stability of secretomes produced under GMP. The findings reported in the current study will help establishing appropriate quality control parameters and specifications related to drug product efficacy and reproducibility for clinical application of human cell-derived, yet cell-free secretomes as biological medicinal products.

## Supplementary information

**Additional file 1: Figure S1.** Cytokine profiling. **Figure S2.** Concentrations of (a) PAI-1 and (b) MMP9 in the secretomes of individual donors. Protein concentrations were determined for secretomes prior to pooling, lyophilization, and terminal sterilization. Each dot represents one donor. Horizontal lines indicate medians. PAI-1, plasminogen activator inhibitor 1; MMP9, matrix metallopeptidase. **Figure S3.** Concentrations of (a) PAI-1 and (b) MMP9 in small batches of PBMC secretomes (12 donors) compared to PBMC^sec^ 1 (open dots) and PBMC^sec^ 2 (black dots). Large batches contain secretomes of 96 – 120 donors. Open dots represent small batches pooled for PBMC^sec^ 1, while black dots denote small batches used for PBMC^sec^ 2. Horizontal lines indicate medians. PAI-1, plasminogen activator inhibitor 1; MMP9, matrix metallopeptidase. **Figure S4.** Potency of secretomes obtained from individual PBMC donors. **Figure S5.** (a) AP-1 promotor activity and (b) HSP27 phosphorylation in small and large secretome batches.

## Data Availability

The datasets analyzed in the current study are available from the corresponding author upon reasonable request.

## Abbreviations

SC: Stem cell
AMI: Acute myocardial infarction
PBMC: Peripheral blood mononuclear cell
PBMC^sec^: Secretome of peripheral blood mononuclear cells
EV: Extracellular vesicle
ATMP: Advanced therapy medicinal product
GMP: Good manufacturing practice
ICH: International Council for Harmonization
SOP: Standard operating procedure
ISO: International Organization for Standardization
Ph. Eur.: Pharmacopoeia Europaea
GY: Green yellow
NTU: Nephelometric turbidity unit
IL-8: Interleukin-8
TGFβ1: Transforming growth factor beta 1
EGF: Epidermal growth factor
MMP9: Matrix metallopeptidase 9
PAI-1: Plasminogen activator inhibitor 1
ELISA: Enzyme-linked immunosorbent assay
GLP: Good laboratory practice
PBS: Phosphate-buffered saline
HUVEC: Human umbilical vein endothelial cells
EBM-2: Endothelial cell growth basal medium
EGM-2: Endothelial cell growth medium
AP-1: Activator protein 1
HSP27: Heat shock protein 27
ANOVA: Analysis of variance
SD: Standard deviation
LOD: Limit of detection
BAFF: B cell activating factor
CD14: Cluster of differentiation 14
ENA-78: Epithelial-derived neutrophil-activating protein 78
IFN-γ: Interferon gamma
MIF: Macrophage migration inhibitory factor
PDGF-AA: Platelet-derived growth factor subunit A
PF4: Platelet factor 4
RANTES: Regulated and normal T cell expressed and secreted
RBP: Retinol binding protein
uPAR: Urokinase-type plasminogen activator receptor
vitamin D-BP: Vitamin D-binding protein
CD31: Cluster of differentiation 31
n.d.: Not determined
