# Supplementary material for: Reproducibility of GMP-compliant production of therapeutic stressed peripheral blood mononuclear cell-derived secretomes, a novel class of biological medicinal products
Source: Stem Cell Res Ther. 2020 Jan 3;11:9. doi: 10.1186/s13287-019-1524-2 (PMC6942406; doi:10.1186/s13287-019-1524-2)
Supplement: Supplementary file 1 — Additional file 1: Figure S1. Cytokine profiling. Figure S2. Concentrations of (a) PAI-1 and (b) MMP9 in the secretomes of individual donors. Protein concentrations were determined for secretomes prior to pooling, lyophilization, and terminal sterilization. Each dot represents one donor. Horizontal lines indicate medians. PAI-1, plasminogen activator inhibitor 1; MMP9, matrix metallopeptidase. Figure S3. Concentrations of (a) PAI-1 and (b) MMP9 in small batches of PBMC secretomes (12 donors) compared to PBMCsec 1 (open dots) and PBMCsec 2 (black dots). Large batches contain secretomes of 96 – 120 donors. Open dots represent small batches pooled for PBMCsec 1, while black dots denote small batches used for PBMCsec 2. Horizontal lines indicate medians. PAI-1, plasminogen activator inhibitor 1; MMP9, matrix metallopeptidase. Figure S4. Potency of secretomes obtained from individual PBMC donors. Figure S5. (a) AP-1 promotor activity and (b) HSP27 phosphorylation in small and large secretome batches. [file 13287_2019_1524_MOESM1_ESM.docx]

Additional file 1

Comparability of GMP-compliant production of therapeutic stressed peripheral blood mononuclear cell-derived secretomes, a novel class of biological medicinal products

Maria Laggner, Alfred Gugerell, Christiane Bachmann, Helmut Hofbauer, Vera Vorstandlechner, Marcus Seibold, Ghazaleh Gouya Lechner, Anja Peterbauer, Sibylle Madlener, Svitlana Demyanets, Dirk Sorgenfrey, Tobias Ostler, Michael Erb, Michael Mildner, Hendrik Jan Ankersmit

**Figure S1.** Cytokine profiling. Analyte and reference annotations on membranes after (a) short and (b) long exposure. (c) Chemiluminescent signals detected on capture antibody-spotted nitrocellulose membranes incubated with different secretome batches or placebo. BAFF, B cell activating factor; CD14, cluster of differentiation 14; EGF, epidermal growth factor; ENA-78, epithelial-derived neutrophil-activating protein 78; IFN-γ, interferon gamma; IL8, interleukin 8; MIF, macrophage migration inhibitory factor; MMP9, matrix metallopeptidase 9; PDGF-AA, platelet-derived growth factor subunit A; PF4, platelet factor 4; RANTES, regulated and normal T cell expressed and secreted; RBP, retinol binding protein; PAI-1, plasminogen activator inhibitor-1; uPAR, urokinase-type plasminogen activator receptor; vitamin D-BP, vitamin D-binding protein; CD31, cluster of differentiation 31.


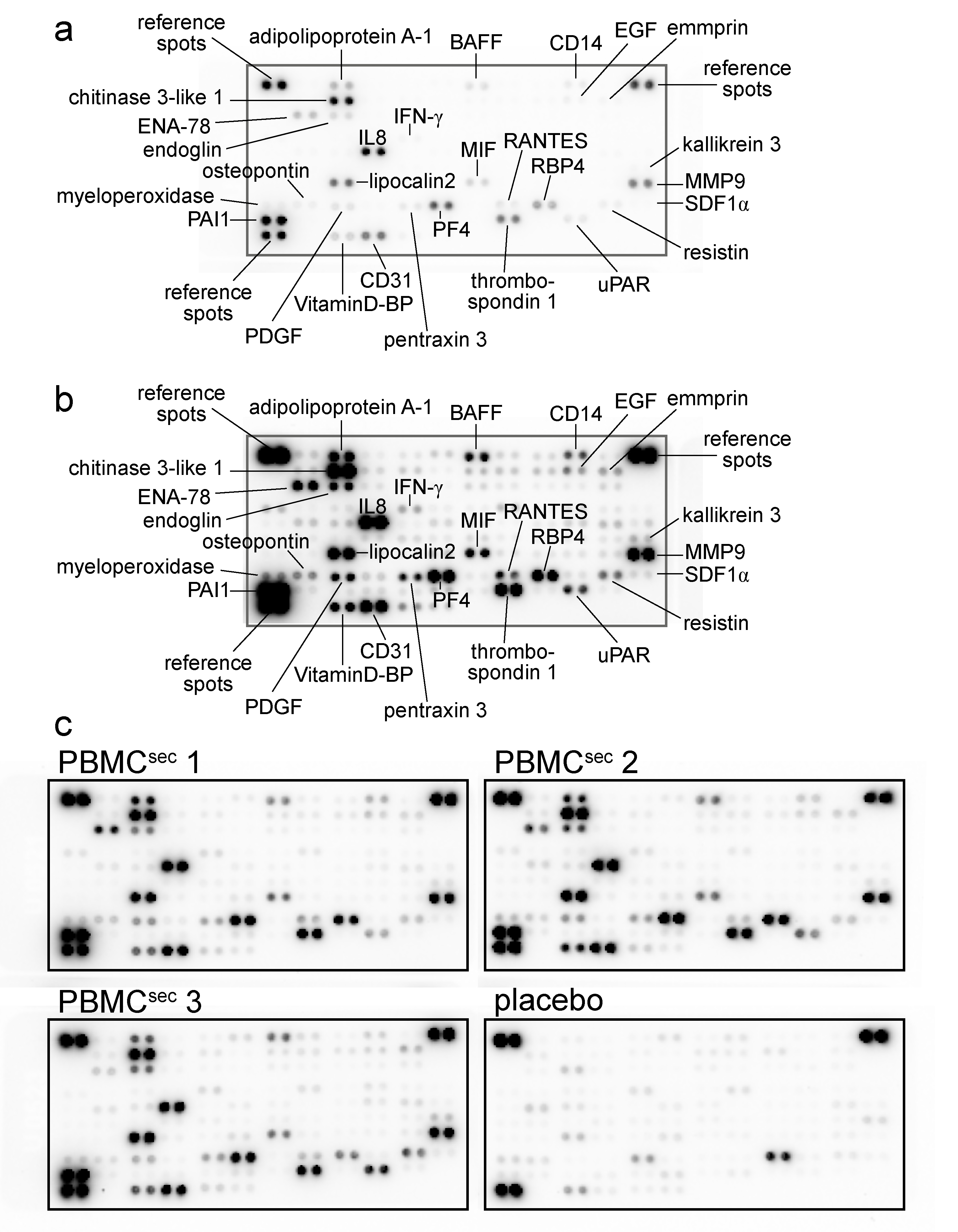


**Figure S2.** Concentrations of (a) PAI-1 and (b) MMP9 in the secretomes of individual donors. Protein concentrations were determined for secretomes prior to pooling, lyophilization, and terminal sterilization. Each dot represents one donor. Horizontal lines indicate medians. PAI-1, plasminogen activator inhibitor 1; MMP9, matrix metallopeptidase.

**Figure S3.** Concentrations of (a) PAI-1 and (b) MMP9 in small batches of PBMC secretomes (12 donors) compared to PBMC^sec^ 1 (open dots) and PBMC^sec^ 2 (black dots). Large batches contain secretomes of 96 – 120 donors. Open dots represent small batches pooled for PBMC^sec^ 1, while black dots denote small batches used for PBMC^sec^ 2. Horizontal lines indicate medians. PAI-1, plasminogen activator inhibitor 1; MMP9, matrix metallopeptidase.

**Figure S4.** Potency of secretomes obtained from individual PBMC donors. Potency was determined for secretomes prior to pooling, lyophilization, and terminal sterilization by measuring (a) AP-1 promotor activity and (b) HSP27 phosphorylation. Each dot represents one donor. Horizontal lines indicate medians. AP-1, activator protein 1; HSP27, heat shock protein 27.

**Figure S5.** (a) AP-1 promotor activity and (b) HSP27 phosphorylation in small and large secretome batches. Drug product potency was assessed in small batches of PBMC secretomes (12 donors) compared to PBMC^sec^ 1 (open dots) and 2 (black dots). Large batches contain secretomes of 96 – 120 donors. Open dots represent small batches pooled for PBMC^sec^ 1, while black dots denote small batches used for PBMC^sec^ 2. Horizontal lines indicate medians. AP-1, activator protein 1; HSP27, heat shock protein 27.
